# Metabolic pathway for a new strain *Pseudomonas synxantha* LSH-7′: from chemotaxis to uptake of *n*-hexadecane

**DOI:** 10.1038/srep39068

**Published:** 2017-01-04

**Authors:** Long Meng, Haoshuai Li, Mutai Bao, Peiyan Sun

**Affiliations:** 1 Key Laboratory of Marine Chemistry Theory and Technology, Ministry of Education, Ocean University of China, Qingdao 266100, China; 2 College of Chemistry & Chemical Engineering, Ocean University of China, Qingdao 266100, China; 3 Key Laboratory of Marine Spill Oil Identification and Damage Assessment Technology, North China Sea Environmental Monitoring Center, State Oceanic Administration, Qingdao 266033, China

## Abstract

Bacteria can use *n*-hexadecane as a carbon source, but it remains incompletely understood whether *n*-hexadecane is transformed into metabolic intermediates prior to cellular uptake or not. We newly isolated a strain identified as *Pseudomonas synxantha* LSH-7′ and conducted chemotaxis experiment of this bacterial strain towards *n*-hexadecane, hexadecanol and hexadecanoic acid with qualitative assays respectively. Furthermore, we described the identification of extracellular alkane hydroxylase and alcohol dehydrogenase activity; acidification of the culture medium; identification of hexadecanoic acid in the culture medium by the GC-MS analysis; and variation concentration of intracellular *n*-hexadecane and hexadecanoic acid. A detailed analysis of the experimental data revealed the chemotaxis of this bacterial strain towards *n*-hexadecane instead of its metabolic intermediates. Our results further suggested that only a fraction of total *n*-hexadecane followed this path, and alkane hydrolase and hexadecanol dehydrogenase were constitutively expressed when grown in the medium of *n*-hexadecane. Most strikingly, we quantitatively investigated the concentration of *n*-hexadecane adsorbed by bacterial chemotaxis. Our findings provided an original insight *n*-hexadecane might be converted to hexadecanoic acid extracellularly before it was taken up across the cell membrane.

Alkanes are found highly abundant constituents of petroleum hydrocarbons are major environmental pollutants as a result of terrestrial and freshwater runoff, refuse from coastal oil refineries, off shore oil production, shipping activities and accidental spills^1^,^2^. Among the alkanes, *n*-hexadecane is a major component. The solubility of *n*-hexadecane in water is 5.21 × 10^−5^ mg L^−1^ at 15 °C and has high partitioning co-efficient 9.1 logKow^3^. Biodegradation of *n*-hexadecane is believed to be a friendly way to environment as well as human beings. For this, it is necessary to know the metabolic mechanisms of *n*-hexadecane.

Singer and Finnerty^4^ observed terminal *n*-hexadecane degradation by *Acinetobacter* HO1-N and *Pseudomonas putida*, and identified five intermediates as *n*-hexadecylhydroperoxide, *n*-hexadecanol, *n*-hexadecyldehyde, *n*-hexadecanoic acid and *n*-hexadecylhexadecanoate in degradation process. However, Whyte *et al*.^5^ reported both terminal and sub-terminal oxidation of *n*-C16 by *Rhodococcus* strain Q15 producing 1-hexadecanol and 2-hexadecanol. Recently, a new insights into alkane metabolism pathways from alkane sensing, chemotaxis, signal transduction, uptake to degradation was put forward by Wang and shao^6^ from the aspect of genes. The underlying mechanisms that control the early responses of bacterial cells to *n*-hexadecane, such as chemotaxis and uptake, remain largely unexplored.

Chemotaxis is defined as the capacity of certain organisms to sense substance concentration gradients and in turn moved towards or away from this gradient^7^,^8^,^9^. Most importantly, alkane chemotaxis had been observed in bacterial isolates of *Flavimonas oryzihabitans* genus^10^, and even the underlying machinery^7^,^11^ had also been identified. Moreover, microorganisms play an important role in the fate of contaminations in the environment, including bioadsorption and passive adsorption^12^. Some researchers had also focused on the surface adsorption and cell uptake of different organic pollutant by microorganism^13^.

About uptake of *n*-hexadecane, there were three types of alkane hydroxylases including methane monooxygenase, membrane-bound non-heme alkane monooxygenase, and cytochrome P450 monooxygenase which degrade short, medium and long chain alkanes^14^. Previous studies had also shown that microorganisms have two ways to access *n*-hexadecane in the culture medium that is adhesion to hydrocarbon droplets (major form of alkanes in water solutions) or biosurfactant assisting emulsification and assimilation of alkanes^15^. However, even if the mechanisms that long-chain fatty acids entered cells had been understood^16^, little is known about whether *n*-hexadecane is converted to metabolic intermediates extracellularly before it is taken up across the cell membrane or not. In addition, the outer membrane (OM) contains many transport proteins^17^,^18^ that mediate the import and export of both small and large molecules. For small hydrophobic molecules, FadL channels^16^ are well known as OM channels with an established role in the uptake of hydrophobic compounds. For large hydrophobic molecules, TonB-dependent receptors are active transporters, requiring cytoplasmic membrane energy to transport their substrates including icons, B_12_, and xenobiotics^18^,^19^.

Therefore, our investigation were to explore metabolic pathway of *n*-hexadecane for newly isolated bacterial strain from chemotaxis to uptake. Firstly, this work was to report the concentration of *n*-hexadecane was absorbed quantitatively in the aspect of bacterial chemotaxis and revealed the chemotaxis of this bacterial strain moved towards *n*-hexadecane instead of its metabolic intermediates. Secondly, to know whether *n*-hexadecane was transformed into metabolic intermediates prior to cellular uptake or not.

## Results and Discussion

### The SEM image, identification and growth of microorganism

**Figure 1:**
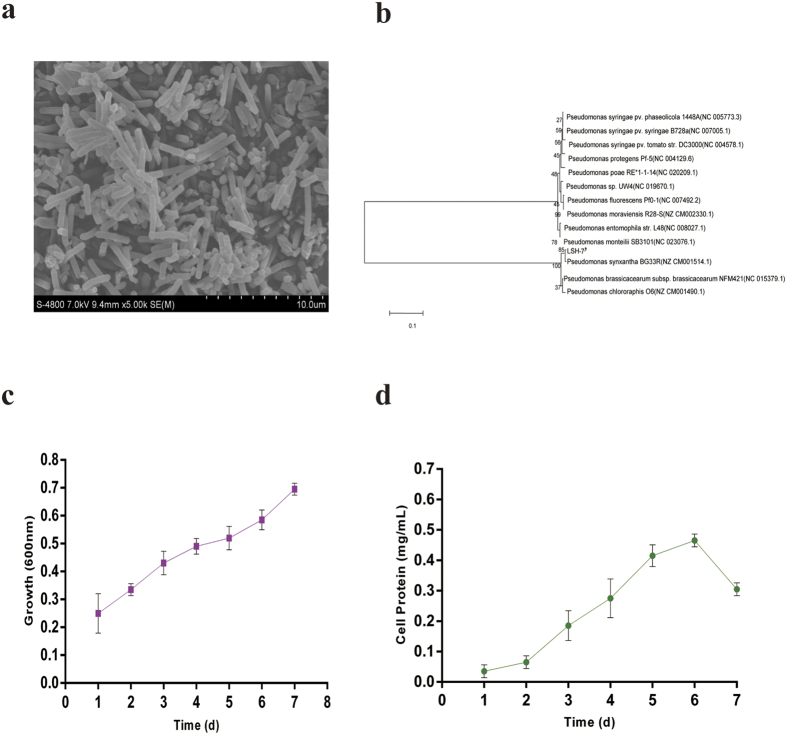
(**a)** Transmission electron microscopy observations of *Pseudomonas synxantha* LSH-7′ cells. **(b)** Phylogenetic relationship based on the 16S rRNA gene sequences between strain *Pseudomonas synxantha* LSH-7′ and species in the Pseudomonas as determined by the neighbor-joiningalgorithm and evaluated by the maximum likelihood and maximum parsimonyalgorithms. **(c)** Growth pattern of bacterial strain with *n*-hexadecane in MSM. The values presented were the averages of two independent experiments. Error bars represented the s.d. **(d)** Bacterial cell protein during the growth of this microbial strain. The values presented were the averages of three independent experiments. Error bars represented the s.d.

The SEM image of this bacteria was shown in Fig. 1(a). It has regular shape of long rod with the particle size from 0.2 to 5 μm. The molecular identification of bacteria was performed amplifying and sequencing the 16S rRNA gene and comparing the sequences to the database of known 16S rRNA sequences. The phylogenetic trees of this strain was shown in Fig. 1(b). The strains selected to structure the phylogenetic trees had above 99% of similarity with *Pseudomonas* genus on the nucleotide sequence. Sequence analysis of the 16S rRNA gene, BLAST sequence comparison and the phylogenetic analysis confirmed that the bacteria was affiliated with *Pseudomonas* genus. Hazen *et al*.^20^ revealed microorganisms that were all classified as *γ*- *Proteobacteria* were significantly enriched in the plume. Among those bacteria, *Pseudomonas* genus were found to be more degrading ability of *n*-hexadecane when compared with others. Tao *et al*.^21^ were also determined to be 96% degradation of *n*-hexadecane by *Pseudomonas aeruginosa* B1, however, 78% by *Acinetobacter* RAG-IB2.

The growth of bacterial strain in MSM medium with 1% *n*-hexadecane was examined (Fig. 1(c)). Bacterial population continued to multiply during 7 days of incubation period. The specific growth rate of this bacterial strain was found to be 0.7 d^−1^ when compared with Jauhari *et al*.^22^ that the specific growth rate of the bacterial strains was found to be 0.56 d^−1^ for E9, 0.48 d^−1^ for BP10, and 0.59 d^−1^ for consortium. Additionally, Kim *et al*.^23^ observed the fastest growth of *Rhodococcus erythropolis* S + 14He when cultured on MSM with 1% *n*-hexadecane.

From Fig. 1(d), cell protein was enhanced by 5 folds during the entire period of bacterial growth. When we compared with the growth of bacteria and the amount of the bacterial protein, two graphs did not seem to be in perfect agreement. The possible reason for such difference (at day 7) was that *n*-hexadecane as the sole carbon and energy of the culture medium was consumed by this bacterial strain and bacterial lysis happened or protein of this bacterial strain became inactivated inevitably. Overall, the amount of the bacterial protein continued to increase with the multiplication of cells in this bacterial strain during incubation period of 7 days. The initial concentration of cell protein varied between 0.02 and 0.05 mg mL^−1^ and then augmented to a maximum of 0.48 mg mL^−1^ at day 6. However, Mishra and Singh^24^ reported the protein content of the bacterial strains ranged from 0.11 to 0.65 mg mL^−1^ at the time of inoculation and Jauhari *et al*.^22^ were determined to be 0.42 mg mL^−1^ in the consortium (BP10 and E9).

### Bacterial chemotaxis and passive adsorption of *n*-hexadecane

Core unit of chemotaxis signaling^7^,^8^,^9^,^10^,^11^ is complicated and diversity in chemotaxis mechanisms are existed among organic pollutants, temperature, or other factors. Kirby^25^ reported chemotaxis-like regulatory systems was an unique roles in diverse bacteria. Ortega-Calvo *et al*.^26^ described chemotactic bacteria with more hydrophobic pollutants, such as high-molecular-mass PAHs. Bharali *et al*.^27^ observed Rhamnolipid from *Pseudomonas aeruginosa* OBP1 could be used as an additive in the formulation of antibiotic and other antimicrobial agents for enhancing the effectiveness of chemotherapeutics.

**Figure 2:**
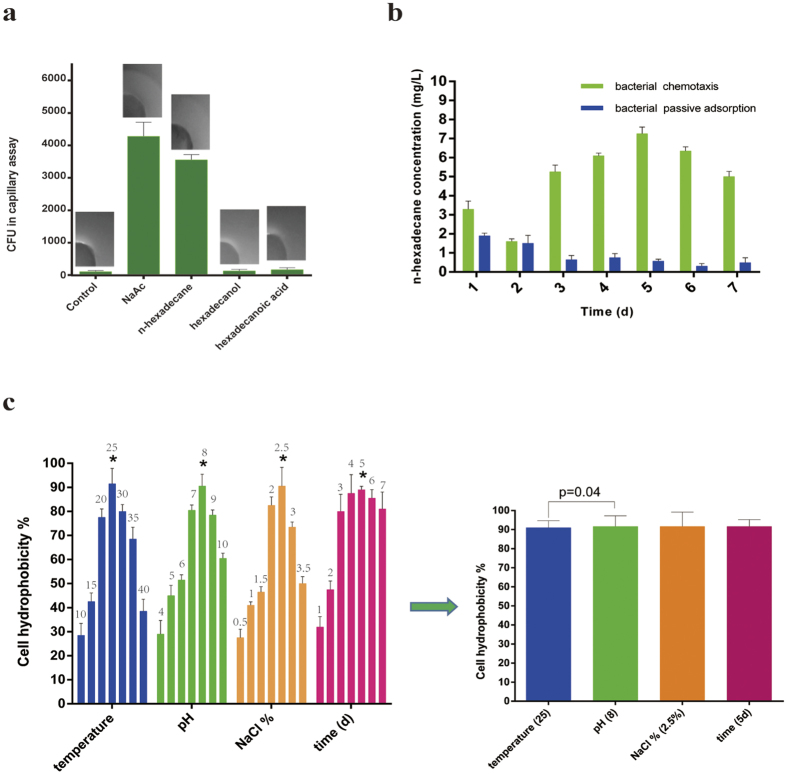
(**a**) Agar plug and capillary assays of bacterial strain chemotactic behavior towards different substrates. The values presented were the averages of three independent experiments. Error bars represented the s.d. (**b**) the concentration of *n*-hexadecane absorbed by bacterial chemotaxis and passive adsorption of *n*-hexadecane by this bacterial strain. The data were presented as the mean of three independent experiments. Error bars represented the s.d. (**c**) Hydrophobicity of microbial strain. (Under the effect of different temperature: 10 °C, 15 °C, 20 °C, 25 °C, 30 °C, 35 °C; under the effect of different pH: 4, 5, 6, 7, 8, 9, 10; under the effect of different NaCl %: 0.5, 1, 1.5, 2, 2.5, 3, 3.5; under the effect of different time: 1d, 2d, 3d, 4d, 5d, 6d, 7d.). Significant difference (p = 0.04) of two factors between temperature and pH. The values presented were the averages of three independent experiments. Error bars represented the s.d.

In our work, it was shown in Fig. 2(a) that significant detectable bacterial chemotaxis was observed towards NaAc and *n*-hexadecane, but no obvious chemotactic behavior was performed in the presence of hexadecanol or hexadecanoic acid. However, Ni *et al*.^28^ showed *Comamonas testosteroni* used a chemoreceptor for tricarboxylic acid cycle intermediates to trigger chemotactic responses towards aromatic compounds. Black *et al*.^29^ was also determined to be that His^110^ of the protein FadL in the outer membrane of *Escherichia coli.* was involved in the binding and uptake of long-chain fatty acids.

Furthermore, to investigate the concentration of *n*-hexadecane absorbed by bacterial chemotaxis, the results were shown in Fig. 2(b). An important information that *n*-hexadecane was easily adsorbed by this bacterial strain. Within the experimental time, the trend of adsorption of *n*-hexadecane was gradually increasing as a whole. Moreover, the maximum of adsorption was found to be 7.2 mg L^−1^ at day 5. Interestingly, *n*-hexadecane approximately decreased to the lowest point at day 2.

To explore passive adsorption of *n*-hexadecane, the adsorption rule by a heat-killed microbial strain along the time was described in Fig. 2(b). The adsorption contents of *n*-hexadecane was gradually decreased on the heat-killed microbial strain. The adsorption of *n*-hexadecane by heat-killed microbial strain was a fast process, however, with the time increasing, the adsorbed *n*-hexadecane were released from dead bacterial strain. This phenomena was also obtained from the bioadsorption behavior of microbial cell for PAHs and alkane which was controlled by the distributional effects with the process of sorption-desorption being reversible^13^,^30^,^31^. Rosenberg and Rosenberg^32^ had shown that the rate of hydrocarbon degradation by bacterial cells was dependent on cell affinity. Cells with high affinity for hydrocarbons utilized *n*-hexadecane more effectively than those with low affinity.

Cell surface hydrophobicity (CSH) is a major factor to determine the adhesion of hydrocarbon to cell surface. In our study (Fig. 2(c)), the bacterial strain had great hydrophobicity and emulsifying ability. It was recorded 90% approximately under the appropriate factors. Mishra and Singh^24^ were determined to be 99.86% CSH in *P. aeruginosa sp.* PSA5, while in *Rhodococcus sp.* NJ2 and *Ochrobactrum sp.* P2, it was found to be 96.4% in MSM enriched with *n*-hexadecane. However, Tebyanian *et al*.^33^ reported cell surface hydrophobicity of 6%, 24% and 29% in *S. maltophilia* strain M2, *S. maltophilia* strain Q1 and *T. tyrosinosolvens* strain Q3, respectively with *n*-hexadecane in the medium. Under the effect of temperature, pH, time and the concentration of NaCl, hydrophobicity of the microbial strain changed strongly. However, the hydrophobicity of the microbial strain that was obtained the high point under four factors were different. In our work, the appropriate temperature, pH, time and the concentration of NaCl were 25 °C, 8.0, 5d, 2.5% respectively.

### Alkane hydroxylase activity

Alkane hydroxylase initiates the aerobic degradation of alkanes by inserting oxygen atoms at the different sites of alkane terminus^5^,^14^,^34^. For examples, Mishra and Singh^24^ observed induction of alkane hydroxylase enzyme in *P. aeruginosa sp.* PSA5 and *Rhodococcus sp.* NJ2 during *n*-hexadecane biodegradation. *Geobacillus thermodenitrificans* NG80-2 can utilize a terminal oxidation pathway for the conversion of long-chain *n*-alkanes (C15-C36) to corresponding primary alcohols^35^.

**Figure 3:**
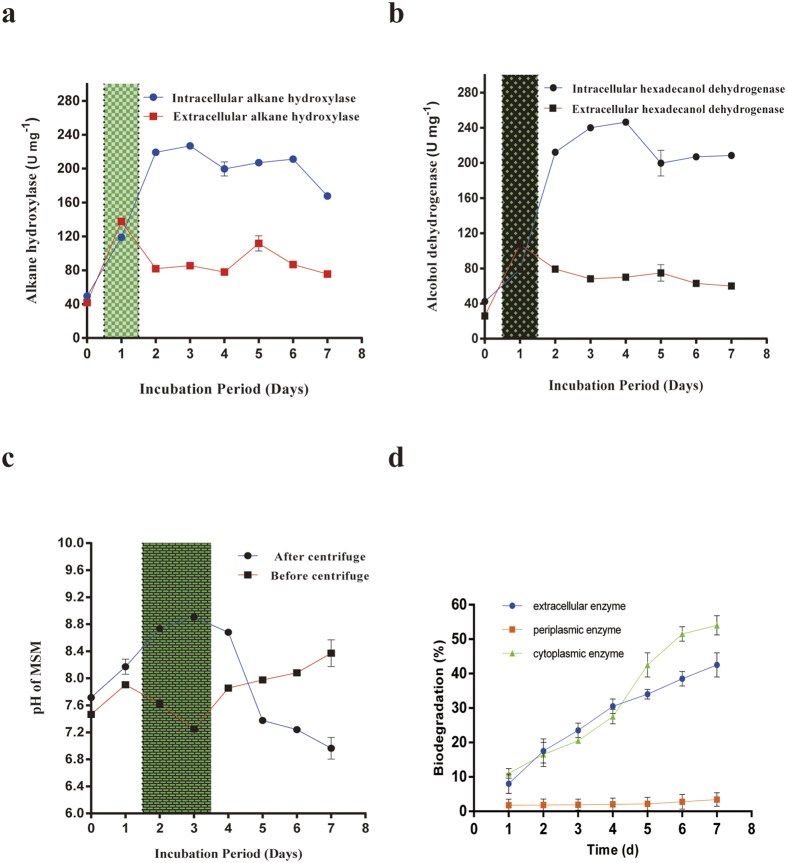
**(a)** Enzyme activities of intracellular alkane hydroxylase and extracellular alkane hydroxylase during biodegradation. **(b)** Analysis of intracellular and extracellular enzymes of hexadecanol dehydrogenase during biodegradation. **(c)** The changes pH of cultured medium. **(d)** Degradation process of *n*-hexadecane by cytoplasmic, periplasmic and extracellular enzymes. All the values presented were the averages of three independent experiments. All error bars represented the s.d.

This study, alkane hydroxylase activity was monitored and shown in the following Fig. 3(a). More importantly, one information was obtained that extracellular alkane hydroxylase activity was higher than the intracellular alkane hydroxylase activity at day 1. That was probably because alkane hydroxylase enzyme was released into extracellular and altered the structure of alkanes of *n*-hexadecane. Peter *et al*.^36^ also reported extracellular enzyme that was capable of catalyzing the selective monooxygenation of diverse organic compounds. Apart from this, the maximum of intracellular alkane hydroxylase activity was found to be 230 U mg^−1^ at day 3 in 7 days incubation period. The intracellular alkane hydroxylase activity increased along with incubation period before day 3 but decreased in other days. However, the extracellular alkane hydroxylase activity had a peak that was 125 U mg^−1^ at day 1 and then kept stabilized from day 2 to day 7.

### Hexadecanol dehydrogenase activity

Alcohol dehydrogenase had been isolated from cell-free extracts of *Pseudomonas sp.* strain 196Aa when grown anaerobically on *n*-alkane^37^. Induction of this enzyme has been also reported in mesophilic, thermophilic and extreme thermophilic microorganisms by Alvarez *et al*.^37^. Abdel-Megeed and Muller^38^ found that alcohol dehydrogenase isolated from *Pseudomonas* genus was able to assimilate and mineralize C10–C22 *n*-alkane as a source of carbon and energy. From this study, the hexadecanol dehydrogenase activity was shown in the following Fig. 3(b). Hexadecanol dehydrogenase activity was completely followed with the alkane hydroxylase activity. The maximum of intracellular alcohol dehydrogenase activity was found to be 245 U mg^−1^ at day 4. More interestingly, alcohol dehydrogenase activity was found higher than alkane hydroxylase. However, Pirog *et al*.^39^ reported alkane hydroxylase activity was higher than alcohol dehydrogenase in *Rhodococcus erythropolis* EK-1 during *n*-hexadecane biodegradation. Furthermore, in our case, we found that alkane hydrolase and hexadecanol dehydrogenase were constitutively expressed when grown on *n*-hexadecane. Inclusions were similar in a *Rhodococcus* genus grown on phenyldecane^39^. Scott and Finnerty^40^ also reported accumulation of hydrocarbons were in inclusion bodies of *n*-alkane degrading bacteria when grown in the presence of *n*-hexadecane. Besides, the intracellular hexadecanol dehydrogenase activity increased along with incubation period before day 4 and decreased in other days. However, the extracellular hexadecanol dehydrogenase activity had a peak that was 115 U mg^−1^ at day 1 and then kept stabilized from day 2 to day 7.

### The pH changes of MSM

The pH of MSM before centrifuge deceased largely at day 2 and day 3 because there existed acid substrate (Fig. 3(c)). In the other days, pH was found to be increased. Mishra and Singh^24^ also observed the pH of the incubation medium was found to range between 7.34 and 7.39, but after 10 days of incubation period, the pH of the medium was slightly decreased to neutrality in the range of 7.0–7.3.

However, the maximum of the pH of MSM after centrifuge was found at day 3 and the pH of MSM after centrifuge increased along with incubation period before day 3 and decreased in other days. It showed there existed the presence of extracellular proteins, and this proteins were considered to be filtered from the supernatant. There was also one information that the pH of MSM after centrifuge drop sharply after day 4. But Jauhari *et al*.^22^ showed the pH of the incubation medium was found to be 7.3. However, after centrifuge, the pH of the medium decreased to 6.5, 5.3 and 5.0 in BP10, E9 and consortium, respectively.

### Periplamic, cytoplasmic and extracellular enzymes in biodegradation of *n*-hexadecane

The success of bioremediation is dependent on the inherent biodegradability of the pollutant^41^. The degradation curves of *n*-hexadecane by periplasmic, cytoplasmic and extracellular enzymes of the microbial strain in different time were presented in Fig. 3(d). Periplasmic enzymes performed little effect on *n*-hexadecane biodegradation in this study. From 1 to 7 d, the degradation rates by the three enzymes were basically different. The extracellular activity was faster than intracellular enzyme activity before 4 d, followed an order of extracellular >cytoplasmic >periplasmic enzyme while slowly than it after 4 d, followed an order of cytoplasmic >extracellular >periplasmic enzyme. However, Deive *et al*.^42^ detected that extracellular lipolytic activity was lower than intracellular enzyme activity. Ye *et al*.^43^ thought that the degradation rates by cells or enzymes exhibited no significant differences and the extracellular enzymes could metabolize contaminants effectively.

### GC-MS analysis and Cell uptake of *n*-hexadecane and hexadecanoic acid

**Figure 4:**
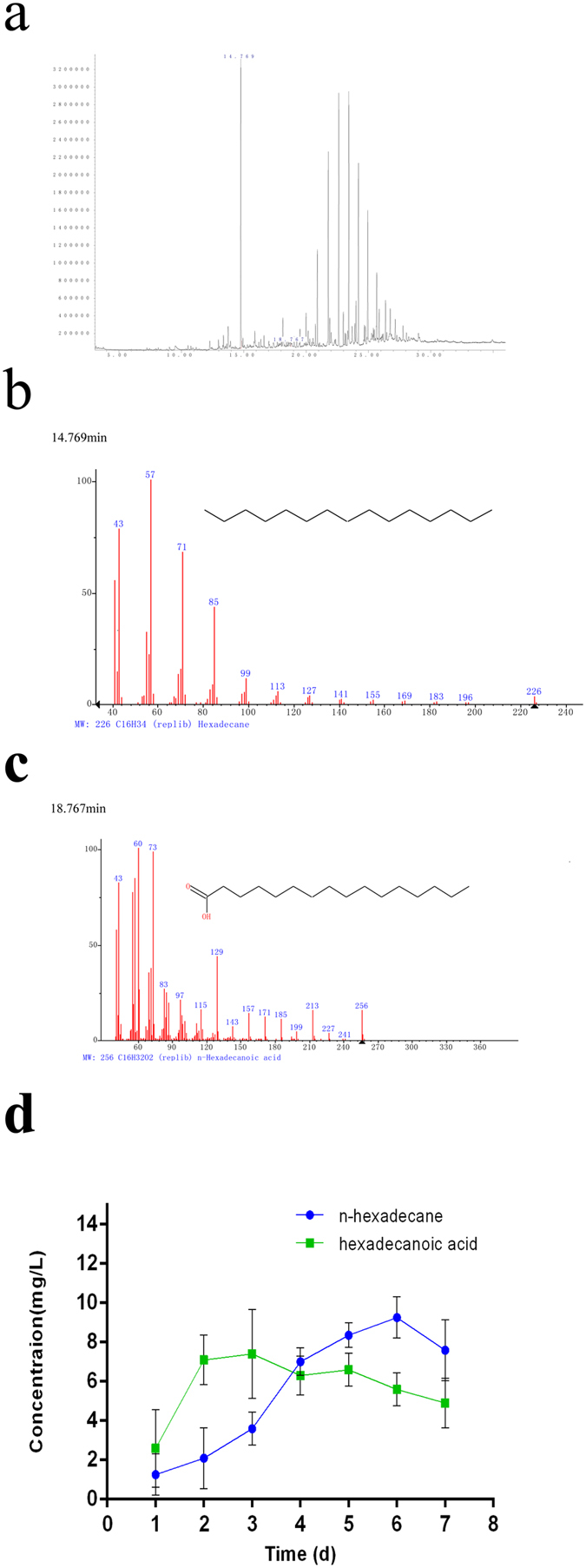
**(a,b,c)** GC-MS of cultured medium at day 1 in 7 days incubation period. **(d)** Cell uptake of *n*-hexadecane and hexadecanoic acid by this bacterial strain. The values presented were the averages of three independent experiments. Error bars represented the s.d.

The results of GC was shown Fig. 4(a), there existed hexadecanoic acid on culture medium at day 1. The retention time of *n*-hexadecane was 14.769 min and the retention time of hexadecanoic acid was 18.767 min. From Fig. 4(b) and (c), the mass spectrum of metabolite was observed at 256 m/z that is identical to hexadecanoic acid. These findings further suggested hexadecanoic acid was found in culture medium at day 1. According to Sanin *et al*.^44^, *Rhodococcus sp.* changed their cell surface properties by altering the composition of cellular fatty acids in the presence of hydrocarbons so that increased lipophilicity could facilitate the uptake, assimilation and import of hydrocarbons. However, this work, we reported a pathway that *n*-hexadecane was transformed into metabolic intermediates prior to cellular uptake. Three possible mechanisms of hydrocarbon uptake^15^ have been proposed. In the first mechanism, microbe directly takes in the hydrocarbon dissolved in the aqueous phase. In the second, the microbial cell takes in the hydrocarbon particle which is dissolved or like-dissolved and much smaller than the cell. In the third, the microbial cell directly contacts with the hydrocarbon which is bigger than the cell, and then absorbs them. Figure 4(d) indicated the cell uptake of *n*-hexadecane and hexadecanoic acid by a live microbial strain along the time to explore the transport rule of cell uptake of substrate. Bouchez-Naïtali *et al*.^45^ also reported the characteristics of alkane uptake and their relevance to a mechanism of interfacial uptake.

This microbial strain had a certain ability of transporting and enriching *n*-hexadecane, and this process might be associated with biodegradation. It proved that only a fraction of total *n*-hexadecane followed this pathway that *n*-hexadecane was transformed into hexadecanoic acid prior to cellular uptake. Cameotra *et al*.^46^ also showed the dispersion of *n*-hexadecane to droplets smaller than 0.22 μm increasing the availability of the hydrocarbon to the degrading organism. However, the concentration of *n*-hexadecane uptake by this strain was lower than bacterial chemotaxis. Additionally, the highest uptake content of *n*-hexadecane and hexadecanoic acid was 8.2 mg L^−1^ at 6 d, 7.3 mg L^−1^ at 4 d respectively.

### An insight on metabolic pathway of *n*-hexadecane

As a step in biodegradation, outer-membrane transport of hydrophobic substrates become an important process for environmental remediation^14^,^18^. In summary, there may be two ways in the cross-membrane transport of *n*-hexadecane. One way is that bacteria seems to respond to growth on alkanes by forming cell aggregates, probably supported by enhanced synthesis of extracellular exopolysaccharides (EPS) and probably following in a quorum-sensing-mediated aggregation process^47^,^48^. Another way is that pollutant may be transferred into cell via lipoprotein directly. The structure of lipoprotein had been studied^16^,^17^,^19^. We believed these findings, for which no previous evidence existed in the literature, were not showed whether *n*-hexadecane was transformed into metabolic intermediates prior to cellular uptake or not. This study went beyond descriptive the concentration of *n*-hexadecane adsorbed by bacterial chemotaxis, bacterial cell hydrophobicity, enzyme activity, pH and GC-MS analysis of the culture medium, and the concentration of intracellular *n*-hexadecane and hexadecanoic acid.

**Figure 5:**
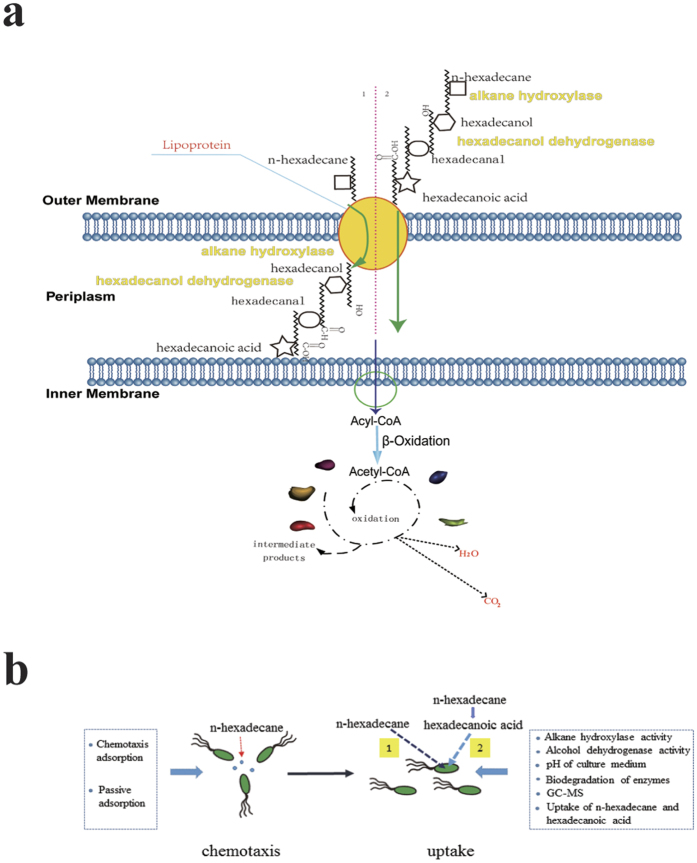
**(a)** The transmembrane network of *n*-hexadecane. 1: the previous research of the transmembrane network of *n*-hexadecane. It directly traverse the outer membrane of bacteria while do not be changed into other substances. 2: the novel insight was proposed. The *n*-hexadecane might be changed into hexadecanoic acid before traverse the outer membrane of bacteria. **(b)** The evidences of the novel insight that *n*-hexadecane might be converted to hexadecanoic acid extracellularly before it was taken up across the cell membrane were presented from chemotaxis to uptake of *n*-hexadecane.

Agar plug and capillary assays (Fig. 2(a)) in this work proved that the chemotaxis of this bacterial strain towards *n*-hexadecane instead of its metabolic intermediates. Cell hydrophobicity (Fig. 2(c)) was of practical important for this. As a first step of oxidation, alkane hydroxylases play an important role in the microbial degradation of alkanes. Under the effect of alkane hydroxylase, alkane will be changed into aliphatic alcohol. Alcohol dehydrogenase is supposed to be an enzyme that have a capacity of oxidizing aliphatic alcohol and also play an important role in degrading alkane aerobically. As was shown in Fig. 3(a), the extracellular alkane hydroxylase activity was higher than the intracellular at day 1, which indicted that alkane hydroxylases were released into extracellular first and altered the structure of *n*-hexadecane later. In Fig. 3(b), alcohol dehydrogenase increased/decreased along with alkane hydroxylase approximately. In Fig. 3(c), because of the occurrence of acid substrates, pH decreased. However, from aspect of pH after centrifuged, we could conclude the biomass might product alkaline substrate for making up for this acid environment. Hence, under the effect of alkane hydroxylase and alcohol dehydrogenase, alkanes were changed into fatty acid, then via the lipid protein channel wall and enter into intracellular of bacteria so as to further oxidization. We proved hexadecanoic acid was existed on culture of medium and GC-MS analysis also further demonstrated this conclusion. Our findings further revealed that there were two ways in the cross-membrane transport of n-hexadecane. Just as Fig. 5(a), only a fraction of total *n*-hexadecane followed the pathway that *n*-hexadecane was transformed into hexadecanoic acid before uptake and the other fraction of total *n*-hexadecane followed the pathway that *n*-hexadecane directly entered into bacterial cell.

We quantitatively investigated the concentration of *n*-hexadecane adsorbed by bacterial chemotaxis. We were aware that the process of bacterial chemotaxis adsorption by a microbial strain was a rapid process. Probably the initial concentrations of the substrates were so high that the substrates were transferred from the high concentration to the low concentration of cell surface. This process might be not associated with biodegradation in a short time. However, it could be presented and the quantity of microbes was increased (Fig. 2(b)) in a long time biodegradation. Moreover, periplasmic enzyme had little effect on crude oil biodegradation while cytoplasmic enzyme and extracellular enzyme both had great effect on it in Fig. 3(d). It could be inferred that alkane hydroxylase and hexadecanol dehydrogenase both played an important role in biodegradation even if they were produced in periplasmic space. The results of above drew attention to indicate that the concentration of *n*-hexadecane absorbed by bacterial chemotaxis was more than uptake by bacteria, however, less than biodegradation by intracellular enzyme.

In summary, a novel insight into transmembrane route was presented in Fig. 5(a) and (b). The proposed pathway was *n*-hexadecane would change into hexadecanoic acid outside of the cell under the effect of alkane hydroxylase and hexadecanol dehydrogenase before entering into bacteria. However, bacterial lysis would inevitably generate negative effect on the experimental results.

## Materials and Methods

### Isolation and screening of bacterial strain

Petroleum sediment was collected from offshore of Qingdao (Loushan river, 36°12’N, 120°20’E) contaminated of crude oil. 10 g of sample was cultivated in 200 mL mineral enrichment medium (MSM) as the sole source of carbon and energy for 20 days at 37 °C on a rotary shaker at 200 r·min^−1^ under aerobic condition. Then 10 mL of culture supernatant was collected and spread on LB agar plates for 3 days. Selection of strains with ability to degrade *n*-hexadecane was conducted in MSM amended with *n*-hexadecane for 7 days at 37 °C on a rotary shaker at 200 r·min^−1^. The MSM contained K_2_HPO_4_ 0.5 g/L, Na_2_SO_4_ 2.0 g/L, NH_4_Cl 1.0 g/L, MgSO_4_∙7H_2_O 0.02 g/L, CaCl_2_ 0.07 g/L and 1.0 ml of trace salt solution per liter. The trace salt solution was defined as 30 mg/L FeCl_3_, 0.5 mg/L CuSO_4_, 0.5 mg/L MnSO_4_∙H_2_O, and 10 mg/L ZnSO_4_∙7H_2_O.

After 7 days of incubation, residual amount of *n*-hexadecane was extracted with hexane (20 mL) thrice and concentrated to 2 mL. Substrate utilization were analyzed using a Gas chromatograph (Agilent 7890 A) with FID detector and a capillary BP5 column (5% phenyl methyl polysiloxane column, 30 m × 0.32 mm × 0.25 μm). Both injection and detector temperature were maintained at 280 °C. Initial oven temperature was maintained 80 °C for 2 min and then increased to 300 °C with 10 °C increase per min^20^,^21^,^22^.

### Microorganisms and Electron microscopy

The strain collected was identified as *Pseudomonas* genus. That was followed the method of 16S rRNA sequencing as described previously^20^.

Bacterial universal primers 515F (GTGCCAGCMGCCGCGGTAA) and 806R (GGACTACHVGGGTWTCTAAT) are used to amplify the V4 region of bacteria l6S rDNA. All PCR reactions are carried out in 30 μL reactions with 15 μL of Phusion^®^ High-Fidelity PCR Master Mix (New England Biolabs); 0.2 μM of forward and reverse primers, and about 10 ng template DNA. Thermal cycling consisted of initial denaturation at 98 °C for 1 min, followed by 30 cycles of denaturation at 98 °C for 10 s, annealing at 50 °C for 30 s, and elongation at 72 °C for 30 s. Finally 72 °C for 5 min. Mix same volume of 1x loading buffer (contained SYB green) with PCR products and operate electrophoresis on 2% agarose gel for detection. Samples with bright main strip between 400–450 bp are chosen for further experiments. PCR products is mixed in equidensity ratios. Then, mixture PCR products is purified with Gene JET Gel Extraction Kit (Thermo Scientific). Sequencing libraries are generated using NEB Next^®^ Ultra™ DNA Library Prep Kit for Illumina (NEB, USA) following manufacturer’s recommendations and index codes are added. The library quality is assessed on the Qubit@ 2.0 Fluorometer (Thermo Scientific) and Agilent Bioanalyzer 2100 system. At last, the library is sequenced on an Illumina MiSeq platform and 250 bp/300 bp paired-end reads are generated.

SEM were carried out as described by Lünsdorf *et al*.^49^. For scanning electron microscopy (SEM), bacterial cells were grown on Permanox slides in ONR7a with either 1.5% *n*-hexadecane as the carbon and energy source.

### Growth of bacterial isolates in MSM with (1%) *n*-hexadecane

The growth of bacterial strain was cultured in sterile containing 10 mL MSM and 1% *n*-hexadecane. All cultures in steriles were incubated in dark in an orbital incubator set at 30 °C and 120 rpm for 7 days. And then samples were collected after 0, 1, 2, 3, 4, 5, 6 and 7 days for analysis. Control sterile without *n*-hexadecane was incubated in the same conditions to serve as reference for the growth (negative control). Growth of bacterial strain in liquid media was measured by UV-visible Spectrophotometer at 600 nm^21^,^22^,^24^,^41^. All tests were done from three independent replicates.

### Protein estimation

For estimation of bacteria cell protein, cells of the bacterial isolated were collected from the MSM with 1% *n*-hexadecane at every one day interval, suspended and washed in potassium phosphate buffer. They were further sonicated and centrifuged at 20,000 rpm at 4 °C for 30 min. The supernatant was subsequently stored at 0 °C and the protein estimation was followed the method of Lowry *et al*.^50^. They were measured at 660 nm by UV-visible spectrophotometer using BSA (bovine serum albumin) as a standard. According to protein standard curve, bacteria cell protein could be estimated. All tests were done from three independent replicates.

### Agar plug assay

Population-scale chemotaxis assays were primarily qualitative, as the agarose-in-plug method^8^. The bacterial strain agar plug assays were done with NaAc (positive control), *n*-hexadecane (positive control), hexadecanol (positive control), and hexadecanoic acid (positive control) respectively^9^,^11^. This strain was grown in Brucella broth plus 10% fetal bovine serum (FBS), a medium called BB10. After growth, the bacteria was collected by low-speed centrifugation, washed and resuspended in a solution of phosphate-buffered saline with 1% dialyzed FBS (PBS1) and warm 0.3% Bacto agar. The final bacterial concentration was ~6 × 10^7^ CFU mL^−1^. This bacterial solution was poured around hard agar plugs, incubated, monitored every 30 min for up to four hours. Plates were then placed at 4 °C for up to 24 hours until images were captured using a digital camera. The hard agar plug composed of PBS1, 2% Bacto agar, and without anything as a negative control.

### Capillary chemotaxis assay

Population-scale chemotaxis assays were primarily qualitative, as the capillary chemotaxis assay method^8^,^9^. The density of bacterial suspensions, prepared as already stated for densitometry assays, was brought up to approximately 5 × 10^7^ CFU mL^−1^. The assays, which followed the classical capillary method, were performed by placing a few drops of these suspensions in a small chamber comprising a U-shaped glass tube between a microscope and coverslip. The chemotactic response was measured by placing the open end of a 1 μL capillary tube containing the chemoeffector solution in the pool of bacterial cells present in the chamber.

After incubation for 30 min at 30 °C, the contents of the capillaries were transferred to tubes of L9 mineral medium. Appropriate dilutions were prepared, and then 0.1 μL samples were spread on plates of 2% agarose in motility buffer (negative control), 2% agarose in motility buffer plus 1% NaAc (positive control), 2% agarose in motility buffer plus 1% *n*-hexadecane (positive control), 2% agarose in motility buffer plus 1% hexadecanol (positive control), and 2% agarose in motility buffer plus 1% hexadecanoic acid (positive control) respectively^7^,^8^,^9^,^10^. Colonies were counted after the plates had been incubated at 30 °C for 16–24 h. The relative error in the determinations by capillary assay was less than 10%.

### The concentration of *n*-hexadecane absorbed by bacterial chemotaxis and passive adsorption

Cell pellets were collected by centrifugation (6000 rpm for 5 min at 4 °C) and washed one time with sterile MSM, two times with respective extractant and the last two times with sterile MSM. The concentrations of *n*-hexadecane that washed solution by this extraction process were considered as the surface adsorption contents by a microbial consortium^13^,^30^,^31^. Samples were measured the concentration of *n*-hexadecane at day 1, 2, 3, 4, 5, 6, 7 respectively. The control were also prepared in the same manner, but without bacterial culture (negative control). All tests were done from three independent replicates. C_1_ represented this concentration of *n*-hexadecane.

Bacterial passive adsorption assay were as the same as above. The heat-killed bacterial cells were obtained by autoclaved at 121 °C for 20 min and the culture medium was centrifuged (6000 rpm for 5 min at 4 °C). The concentration of n-hexadecane was determined. The dry weight of heat-killed bacterial cells was measured by oven-dried at 60 °C until it reached a constant. All tests were done from three independent replicates. The control were also prepared in the same manner, but without bacterial culture (negative control). C_2_ represented this concentration of *n*-hexadecane (positive control).

The concentration of *n*-hexadecane is measured by Agilent Gas Chromatograph (7890A) with FID using capillary BP5 column (5% phenyl methyl polysiloxane column, 30 m × 0.32 mm × 0.25 μm). Both injection and detector temperature are maintained at 280 °C. Initial oven temperature is maintained 80 °C for 2 min and then increase to 300 °C with 10 °C increase per min. Bacterial chemotaxis was measured and calculated by the formula as below:


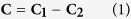


where, **C** = the concentration of *n*-hexadecane absorbed by bacterial chemotaxis; **C**_**1**_ = the concentration of *n*-hexadecane absorbed bacterial surface; **C**_**2**_ = the concentration of *n*-hexadecane absorbed by bacterial passive adsorption.

### Measurement of surface hydrophobicity of bacteria

The BATH assay^13^ is used to measure the cell surface hydrophobicity of bacterial strains to *n*-hexadecane as a substrate. It represents the percent adherence of bacterial cell to hydrophobic substrates. The percentage of cells adhering to hydrocarbons is calculated by the following equation:


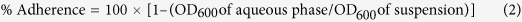


Cell surface hydrophobicity was measured for 7 days of incubation with *n*-hexadecane. All tests were done from three independent replicates. The control were also prepared in the same manner, but without bacterial culture (negative control).

### Measurement of alkane hydroxylase activity

According to Mishra and Singh^24^ and Jauhari *et al*.^22^, aqueous phase of MSM is collected, centrifuged at 8000 rpm. Intracellular and extracellular enzymes (positive control that relative to another) are effectively separated. The supernatant is used for extracellular enzyme activity, and cells are harvested are prepared for intracellular enzyme activity.

Intracellular alkane hydroxylase is resuspended in 20 mmol L^−1^ Tris-HCl buffer (pH 7.4), disrupted using ultrasonic disintegrator (Fisher model 300), and centrifuged for 10 min at 8000 rpm. The cell-free supernatant is assayed for alkane hydroxylase activity. The reaction mixture contain 20 mmol L^−1^ Tris-HCl and 0.15% CHAPS buffer (pH 7.4), 0.1 mmol L^−1^ NADH, 10 μL of *n*-hexadecane solution (1% in 80% DMSO), and 50 μL enzyme in 1 mL volume. To start the reaction, 10 μL of *n*-hexadecane solution was added to the reaction mixture.

Seemly, extracellular enzyme activity is performed better followed by the concentration of supernatant and appropriate temperature that can ensure NADH would be available extracellularly. The reaction mixture contain 20 mmol L^−1^ Tris-HCl and 0.15% CHAPS buffer (pH 7.4), 0.1 mmol L^−1^ NADH, 10 μL of n-hexadecane solution (1% alkanes of crude oil in 80% DMSO), and 50 μL enzyme in 1 mL volume. To start the reaction, 10 μL of n-hexadecane solution is added to the reaction mixture. The control were also prepared in the same manner, but NaCl solution (negative control). One unit of alkane hydroxylase activity was corresponded to amount of enzyme which oxidized 1 μmol NADH per min.

As shown in Fig. S1, the quadratic function of standard curve of NADH concentration was:


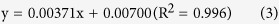


### Measurement of hexadecanol dehydrogenase activity

Measurement of hexadecanol hydroxylase activity can be performed by Jadhav *et al*. (2015)^22^. A queous phase of MSM is collected, centrifuged at 8000 rpm. Intracellular and extracellular enzymes (positive control that relative to another) are effectively separated. The supernatant is used for extracellular enzyme activity and cells are harvested are prepared for intracellular enzyme activity.

Assay mixture (800 μL) consist of 0.05 M sodium pyrophosphate buffer of pH 8.8 (260 μL), 50 mmol L^−1^ hexadecanol (220 μL), 15 mmol L^−1^ NAD (300 μL) and reaction is started immediately by addition of enzyme solution (20 μL, 0.1 mg/mL). Reduction of NAD to NADH is followed for 4 min by taking absorbance at 340 nm. The assay mixture for the blank (800 μL) consisted of 0.05 mol L^−1^ sodium pyrophosphate buffer of pH 8.8 (260 μL), 50 mmol L^−1^ hexadecanol (220 μL), 15 mmol L^−1^ NAD (300 μL) and distilled water (20 μL). One unit of hexadecanol hydroxylase activity is corresponded to amount of enzyme which reduce 1 μmol NADH per min. The control were also prepared in the same manner, but NaCl solution (negative control). Enzyme activity was measured by an increase in absorbance at 340 nm of NADH on spectrophotometer (Fig. S1). Seemly, extracellular enzyme activity was performed better followed by the concentration of supernatant and appropriate temperature that could ensure NAD would be available extracellularly.

### The pH changes of MSM

The pH of aqueous phase is measured by acidity meter (PB-10, from Beijing equity instrument). The pH of MSM before centrifuge and MSM after centrifuge (positive control that relative to another) were measured respectively.

### Biodegradability of periplasmic, cytoplasmic and extracellular enzymes

Osmotic shock method is employed to collect the enzymes in different cell parts^30^,^42^. The degradation medium containing *n*-hexadecane (20 mg L^−1^) has inoculated with 10% the bacteria and incubated at 30 °C with shaking at 120 rpm for 3 d. The cell pellet P_1_ is harvested by centrifugation (6000 rpm for 10 min at 4 °C). The supernatant is filtered (0.45 μm) for removal of microorganisms was S_1_. Then the pellet P_1_ is washed 2 times with cold triple-distilled water is resuspended into 10 mL Tris-HCl (pH = 8, 10 mmol L^−1^), centrifuged (6000 rpm for 10 min at 4 °C) and filtered (0.45 μm, S_2_). The pellet P_2_ is resuspended into 10 mL sucrose solution (25%), shook at 30 °C, 120 rpm for 10 min, centrifuged (10,000 rpm for 10 min at 4 °C) and filtered (0.45 μm, S_3_). The pellet P_3_ is washed 2 times with cold triple-distilled water is resuspended into 10 mL cold triple-distilled water, shook in an ice bath for 10 min, centrifuged (13,000 rpm for 10 min at 4 °C) and filtered (0.45 μm, S_4_). After the pellet P_4_ is resuspended into 10 mL Tris-HCl (pH = 7.4, 10 mmol L^−1^), cell disruption is carried out in ice bath using a ultrasonic processor for 3 min, followed by centrifugation (15,000 rpm for 20 min at 4 °C) and filtration (0.45 μm, S_5_). The filtrate S_1_, S_2_, S_3_, S_4_ and S_5_ are collected and saved at 4 °C, respectively.

Periplasmic (S_4_), cytoplasmic (S_5_) and extracellular enzyme (mixed S_1_, S_2_ and S _3_) (10%, v/v) is inoculated into 20 mg L^−1^ of *n*-hexadecane and cultivated at 30 °C with shaking at 120 rpm for 1, 2, 3, 4, 5, 6 and 7 d, respectively. All tests were done from three independent replicates. The control (NaCl) is performed under the same conditions. Biodegradation rate is measured by GC as above.

### Gas chromatography-mass spectrometry (GC-MS) analysis

GC-MS analysis^24^,^41^ is done on culture medium and affirm whether the *n*-hexadecane change into hexadecanoic acid or not. The injection port of a 7890A model Gas Chromatographer (Agilent Technologies, Inc., Santa Clara, CA) with a 30 m × 0.25 mm DB-5 capillary column (Agilent Technologies, Inc.) coupled to an Agilent MS 5975C model Mass Spectrometer are used. The oven temperature program was 100 °C for 2 min, 150 °C for 4 min, and a ramp to 250 °C at a rate of 4 °C min^−1^. One microliter of sample was injected with a 1:10 split ratio. Peak identification was achieved by comparison with internal standards and to the NIST Mass Spectral Database. The control were also prepared in the same manner, but without bacterial culture (negative control).

### Cell uptake of *n*-hexadecane and hexadecanoic acid

The bacterial cells are suspended into 10 mL Tris-HCl (pH = 7.4, 10 mmol L^−1^) and broken up ultrasonically in an ice bath with 10s pulse and 10s pause for a total period of 3 min. After centrifugation (7000 rpm for 10 min at 4 °C), supernatant is considered as the contents of cell uptake of *n*-hexadecane and hexadecanoic acid. Samples were measured the substrate concentration at day 1, 2, 3, 4, 5, 6 and 7, respectively^41^,^42^,^43^. All tests were done from three independent replicates. The control were also prepared in the same manner, but without bacterial culture (negative control).

The concentration of *n*-hexadecane is measured by GC as above and the concentration of hexadecanoic acid is measured by Titration method^44^,^45^.

## Additional Information

**How to cite this article**: Meng, L. *et al*. Metabolic pathway for a new strain *pseudomonas synxantha* LSH-7': from chemotaxis to uptake of *n*-hexadecane. *Sci. Rep.* **7**, 39068; doi: 10.1038/srep39068 (2017).

**Publisher's note:** Springer Nature remains neutral with regard to jurisdictional claims in published maps and institutional affiliations.

## Supplementary Material

Supplementary Information
