# Supplementary material for: Metabolic pathway for a new strain Pseudomonas synxantha LSH-7′: from chemotaxis to uptake of n-hexadecane
Source: Sci Rep. 2017 Jan 4;7:39068. doi: 10.1038/srep39068 (PMC5209730; doi:10.1038/srep39068)
Supplement: Supplementary Information [file srep39068-s1.pdf]

Scientific Reports

Metabolic pathway for a new strain *Pseudomonas synxantha* LSH-7': from chemotaxis to uptake of *n*-hexadecane

Long Meng[1,2], Haoshuai Li[1,2], Mutai Bao[1,2,*] & Peiyan Sun[3]

[1] *Key Laboratory of Marine Chemistry Theory and Technology, Ministry of Education, Ocean University of China, Qingdao 266100, China*

[2] *College of Chemistry & Chemical Engineering, Ocean University of China, Qingdao 266100, China*

[3] *Key Laboratory of Marine Spill Oil Identification and Damage Assessment Technology, North China Sea Environmental Monitoring Center, State Oceanic Administration, Qingdao 266033, China*

[*] Corresponding author: mtbao@ouc.edu.cn (M. Bao), Tel/Fax: +86-532-66782509, E-mail: mtbao@ouc.edu.cn ,

Tel/Fax: +86-532-66782509.

Supplementary information

Figure S1

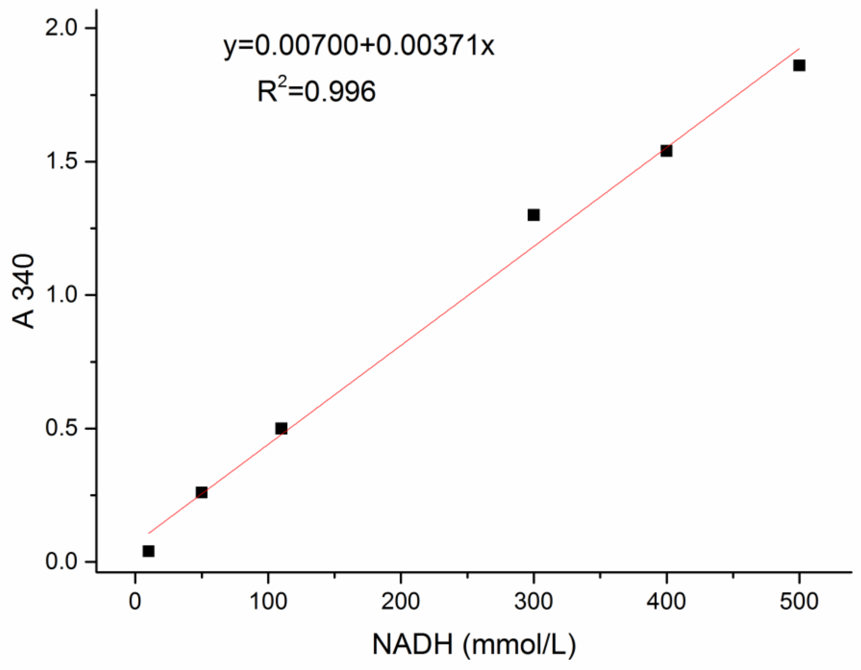

Fig. S1. The calibration curve between absorbance (A340) and the concentration of NADH (mmol/L).
